# Degradation of four pesticides in five urban landscape soils: human and environmental health risk assessment

**DOI:** 10.1007/s10653-022-01278-w

**Published:** 2022-05-11

**Authors:** Islam Md Meftaul, Kadiyala Venkateswarlu, Prasath Annamalai, Aney Parven, Mallavarapu Megharaj

**Affiliations:** 1 Global Centre for Environmental Remediation (GCER), College of Engineering, Science and Environment, The University of Newcastle, ATC Building, University Drive, Callaghan, NSW 2308 Australia; 2 Department of Agricultural Chemistry, Sher-e-Bangla Agricultural University, Dhaka, 1207 Bangladesh; 3 Formerly Department of Microbiology, Sri Krishnadevaraya University, Anantapuramu, 515003 India; 4 Cooperative Research Centre for Contamination Assessment and Remediation of the Environment (CRC CARE), The University of Newcastle, Callaghan, NSW 2308 Australia

**Keywords:** Urban soils, Pesticide degradation, Half-life, Human health risk, Environmental hazard

## Abstract

Pesticides are the most cost-effective means of pest control; however, the serious concern is about the non-target effects due to their extensive and intensive use in both agricultural and non-agricultural settings. The degradation rate constant (*k*) and half-life (DT_50_) of four commonly used pesticides, glyphosate, 2,4-D, chlorothalonil and dimethoate were determined in five Australian urban landscape soils, with varying physicochemical characteristics, to assess their environmental and human health risks. The *k* values (day^−1^) for the selected pesticides were inversely proportional to those of organic carbon (OC), silt, clay and Fe and Al oxides, and directly proportional to pH and sand content in soils. In contrast, the calculated values of DT_50_ (days) of all the four pesticides in five soils positively correlated with OC, clay, silt and oxides of Fe and Al, whereas soil pH and sand content exhibited a negative correlation. The calculated values of environmental indices, GUS and LIX, for the selected pesticides indicate their potential portability into water bodies, affecting non-target organisms as well as food safety. The evaluation for human non-cancer risk of these pesticides, based on the calculated values of hazard quotient (HQ) and hazard index (HI), suggested that exposure of adults and children to soils, contaminated with 50% of initially applied concentrations, through ingestion, dermal and inhalation pathways might cause negligible to zero non-carcinogenic risks. The present data might help the stakeholders in applying recommended doses of pesticides in urban landscapes and regulatory bodies concerned in monitoring the overall environmental quality and implementing safeguard policies. Our study also clearly demonstrates the need for developing improved formulations and spraying technologies for pesticides to minimize human and environmental health risks.

**Graphic abstract:**

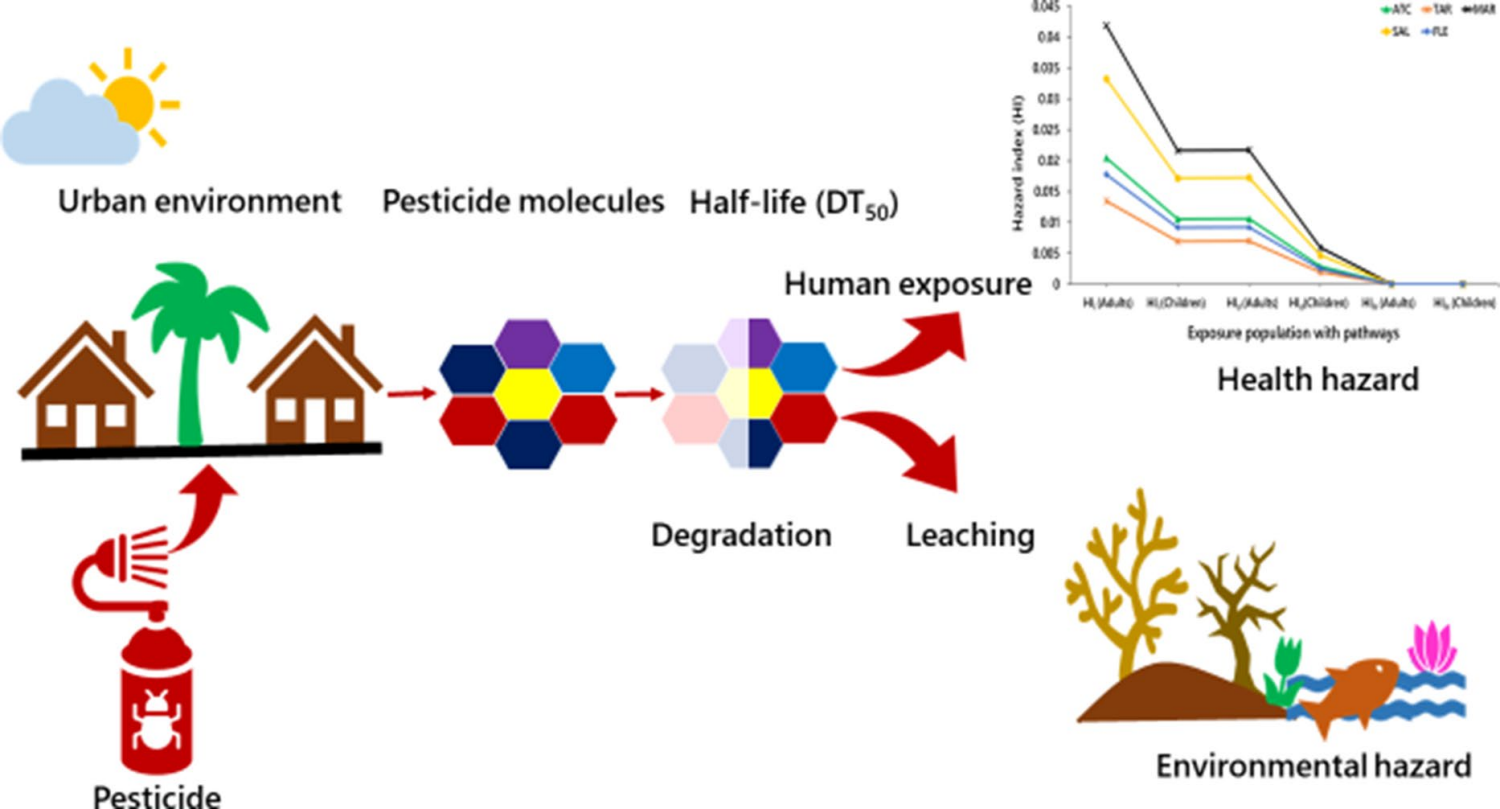

**Supplementary Information:**

The online version contains supplementary material available at 10.1007/s10653-022-01278-w.

## Introduction

Globally, an average of three million tons of synthetic pesticides is applied on annual basis in agricultural and non-agricultural activities, resulting in unintentional toxicity to non-target biota (Fernandes et al., 2020; Shukla et al., 2006). Intensive and improper use of these agrochemicals leads to soil and water contamination causing imminent environmental and health hazards (Ahmad et al., 2019; Fernandes et al., 2020; Karimi et al., 2021). The acute human health hazards like headaches, abdominal pain, nausea, vomiting, dizziness, skin and eye irritation, etc. are due to short-term exposure to pesticides (Miah et al., 2014). Besides being possible human carcinogens, mutagens and acetylcholinesterase inhibitors, most of these pesticides cause chronic toxicities like reproductive toxicity, genotoxicity, endocrine disruption, kidney damage, metabolic alterations, liver and bladder toxicity, gastrointestinal problems, etc. (EPA, 2017; Huang et al., 2019; Ramakrishnan et al., 2018; Zhang et al., 2015). Based on the joint report of UNEP and WHO, approximately 200,000 people die throughout the world, and roughly 3 million are poisoned every year because of pesticides (Meftaul et al., 2020a; Pope et al., 1994). Though bulk amounts of pesticides are used in high-income countries, a vast majority (95%) of pesticide toxicity cases occur in developing nations due to lack of awareness, misuse, improper handling, etc. (Parven et al., 2021; Yadav et al., 2015).

Nevertheless, several pesticides are extensively used at higher doses in urban agricultural and non-agricultural settings that may result in increased contamination (Pino & Peñuela, 2011). In general, herbicides act as plant cell membrane disrupters besides being inhibitors of photosynthesis, pigment biosynthesis, lipid biosynthesis and amino acid synthesis, while fungicides inhibit protein biosynthesis, ergosterol biosynthesis and mitochondrial respiration, and insecticides mostly affect muscle and nerves, energy production and eventually growth and development of insect (Lushchak et al., 2018). The use of pesticides must ensure human and ecological safety from both the parent compounds and their hazardous metabolites (Arias-Estévez et al., 2008). Indeed, degradation is a vital process of reducing the levels of pesticide residues in soil (Hu et al., 2018; Quintero et al., 2005). The process of pesticide transformation in soil mostly occurs through microbial degradation and abiotic degradation (hydrolysis, photolysis and oxidation), wherein biodegradation is dependent on the structure and physicochemical characteristics of both soils and pesticides (Cycoń et al., 2017; Singh et al., 2006). Pesticide degradation in soils yields metabolites of varying toxicities or sometimes results in complete mineralization (Singh et al., 2006). However, the environmental fate of pesticides depends on how strongly they are bound to the soil matrix and the degradation rate (Arias-Estévez et al., 2008). Some pesticides that are insoluble in water have a tendency to get strongly sorbed to soil particles making them relatively unavailable for biodegradation and persist for longer periods thereby adversely affecting soil biology (Purnomo et al., 2011; Wang et al., 2013). The potential detrimental impacts of pesticides in soil include antagonistic effects on the population of soil microflora, alterations in activities of soil enzymes, changes in nitrogen balance of soil by inhibiting N_2_ fixation and ammonification, hostile effects on mycorrhizal symbiosis and nodulation in legumes, and eventually affecting soil fertility and plant growth (Das et al., 2016; Cycoń et al., 2017; El Alfy & Faraj, 2017).

In urban agricultural activities that include home gardens, flowering and ornamental plants, large trees and non-agricultural settings such as golf courses, domestic lawns, garages, driveways, footpaths and other pavements, pesticides are extensively applied for controlling undesirable noxious weeds, woody species and other invasive species (Meftaul et al., 2020b). Moreover, the application rate of pesticides in urban landscapes worldwide is tenfold higher than in agricultural farms (USFWS, 2000). For example, the annual usage of pesticides on lawns is approximately 80 million pounds of active ingredients in the USA, wherein home and garden usage accounts for 15, 10 and 8% of total insecticides/miticides, fungicides and herbicides, respectively (Bush, 2018; Grube et al., 2011). The range of organochlorine residues reported in urban soils was 0.0002–1243.68 mg kg^−1^ in Brazil (Fernandes et al., 2020), 0.01–62.80 µg kg^−1^ in Central India (Kumar et al., 2018), limit of detection (LOD)–182 ng g^−1^ in Novi Sad, Serbia (Škrbić et al., 2017), 1.40–60.0 ng g^−1^ in Nowshera, Pakistan (Zehra et al., 2015), LOD–889.0 ng g^−1^ in Romania (Ene et al., 2012), 4.0–1018.30 ng g^−1^ in Europe (Holoubek et al., 2009), 0.03–1282.58 ng g^–1^ (Yang et al., 2010) and 0.32–136.43 ng g^–1^ (Yang et al., 2012) in Beijing, China. Indeed, the above concentrations are considerably greater than reported in agricultural soils, thus representing the higher risk factor in the urban areas (Fernandes et al., 2020). Consequently, children and pets could easily be exposed to a pesticide from the urban landscape, resulting in both acute and chronic health effects (Hoover, 2005).

The most frequently used pesticides in the urban environment include glyphosate (N-(phosphonomethyl) glycine), 2,4-D (2,4-dichlorophenoxyacetic acid), dimethoate (O,O-dimethyl-S-[2-(methylamino)-2-oxoethyl] dithiophosphate) and chlorothalonil (2,4,5,6-tetrachlorobenzene-1,3-dicarbonitrile) to control pests and diseases (Meftaul et al., 2020a). In fact, urban soil properties are considerably altered due to anthropogenic activities and are distinct from other natural or agricultural soils (Bullock & Gregory, 2009; Yu et al., 2012). The modifications that occur in the soil during urban infrastructure development are ped breakdown, micropore collapse and increase in bulk density, all of which alter the microbial activities and biomass, and soil organic matter (OM) quality (Pouyat et al., 2002; Scharenbroch et al., 2005). The application of nutrients and OM to soil greatly influences the activity and structure of fungal and bacterial populations through increased metabolism, consequently affecting pesticide degradation (Marin-Benito et al., 2012). Although pesticide application in the urban environment has been intensive throughout the globe (Eigenbrod & Gruda, 2015; Okada et al., 2020), the degradation and risks of pesticide exposure are poorly understood in urban landscape soils. Generating such detailed knowledge on the degradation of most frequently used pesticides, such as glyphosate and 2,4-D (herbicides), dimethoate (insecticide) and chlorothalonil (fungicide), in urban landscape soils is greatly warranted in assessing their potential hazards in human and environmental health. Therefore, the current novel investigation aimed to determine the half-life (DT_50_) of the above four extensively used pesticides in five urban landscape soils with varying physicochemical characteristics for assessing the environmental and human health risks.

## Materials and methods

### Chemicals

Four pesticides, viz. glyphosate, 2,4-D, chlorothalonil and dimethoate of ≥ 98% purity, were obtained from Merck. Acetonitrile and methanol (LC–MS grade), and certified standard chemicals (MS grade, ≥ 99.9% purity) like CaCl_2_, KCl, formic acid, acetic acid, phosphoric acid, ammonium acetate and ammonium formate were also purchased from Merck. The commercial formulations of the selected pesticides, glyphosate (100 g L^‒1^ isopropylamine salt) and 2,4-D amine 625 (625 g L^‒1^ dimethylamine and diethanolamine salts), chlortan 720 (720 g L^‒1^ chlorothalonil), dimethoate (400 g L^‒1^ dimethoate), were obtained from CRT Raymond’s Warehouse (suppliers of pesticide products in Australia). Aliquots of 1000, 160, 133.33 and 250 μL of the commercial formulations were diluted to 1.0 L with Milli-Q water to obtain 100 mg L^‒1^ stock solution of glyphosate and 2,4-D, chlorothalonil and dimethoate, respectively. Then, 1.0 mL of each pesticide solution (100 mg L^‒1^) was applied to 20 g soil to provide a final concentration of 5 mg L^‒1^.

### Soil sampling and analysis

Five fresh urban soils, dedicated initially for growing vegetables, lawn grass, flowers, ornamental plants, etc., were obtained from the surface (0–15 cm) in the Hunter region, Australia (Fig. S1). Five bulk soil samples were collected randomly from each location and mixed thoroughly to obtain a composite sample. The soil samples were assigned with specific IDs (Table S1), air-dried, sieved through a 2-mm-diameter mesh and stored at 21 ± 1 °C. The physicochemical characteristics of each urban soil were determined using triplicate (*n* = *3*) samples (Table S1). The hydrometer method (Gee & Or, 2002) was followed to determine the soil texture, in terms of % sand, silt and clay. Electrical conductivity (EC) and soil pH were determined using a pH meter (Laqua, Horiba Scientific) in a suspension containing 5-g soil and 25 mL Milli-Q water. The percentage of soil OC was determined in a LECO analyser equipped with a non-dispersive infrared detector (LECO Corporation, Australia). Fe and Al were extracted from 0.50 g soil samples using 5 mL aqua regia solution, digested in a Microwave Digestion System (MARS 6™, USA) and measured by ICP-OES (PerkinElmer Pvt Ltd, Singapore). Following Fourier transform infrared spectroscopy (FT-IR, Agilent Technologies, USA), the functional groups of soil organic matter were analysed (Fig. S2). The soil mineral composition was determined using peaks from X-ray diffractometer (PANalytical, the Netherlands). The physicochemical characteristics of the five selected urban landscape soils are shown in Table S1.

### Pesticide degradation experiments

A set of 20-g portions of each soil, contained in centrifuge tubes covered with perforated aluminium foil, was stored in dark at 21 ± 1 °C for 30 days, and moisture content of the soil was maintained at 15% on weight basis before the start of degradation studies (Hiller et al., 2010). Soils were then spiked with 1.0 mL of 100 mg L^‒1^ aqueous solutions prepared from commercial formulations of the selected pesticides to provide a final pesticide concentration of ∼5 mg kg^‒1^ active ingredient in the soil matrix. The soils were thoroughly mixed and allowed for two h for equilibration. The soil moisture content was maintained at 70% of water-holding capacity by the addition of appropriate aliquots of Milli-Q water and incubated in dark at 21 ± 1 °C to check for microbial degradation (Hiller et al., 2010). Another set of tubes with soil-applied pesticides was incubated at ‒20 °C in a cold room to check for chemical degradation. A set of tubes that received no pesticides served as control. Duplicates of each soil sample (10 g) were taken at 0, 3, 7, 10, 15, 20, 30, 45, 60, 90, 120 and 150 days of incubation to extract and determine the residues of pesticides remained in soil samples. Pesticides were extracted by shaking the soil samples with 50 mL of methanol for three h. After centrifuging at 2750 × *g* for 15 min, 2 mL aliquots from supernatants were used for the analysis of glyphosate, 2,4-D and dimethoate using LC–MS, and chlorothalonil following LC-DAD system.

### Analytical methods

Concentrations of glyphosate, 2,4-D and dimethoate in aliquots of the extracts were determined using an LC–MS (Agilent 1260/6150B, Agilent Technologies, USA) fitted with Zorbax Eclipse plus C18 column of 4.6 × 150 mm and 3.5 μm dia (Agilent Technologies, USA). The set parameters for single quadrupole mass spectrometer were as follows: oven temperature 60 °C (glyphosate) and 35 ℃ (2,4-D and dimethoate), capillary voltage of 4000 V, drying gas flow of 12.0 mL min^‒1^ at 300 ℃, 100 V fragmentor voltage, 35 psi nebuliser pressure and sheath gas flow of 3.0 mL min^−1^ at 150 °C, negative mode with SIM ion 124 → 168 amu (glyphosate), 219 → 221 amu (2,4-D) and positive mode with 125 → 230 amu (dimethoate). For analysis of glyphosate, mobile phases used were 1% aqueous acetic acid (A) and 1% acetic acid in methanol (B) with a flow rate of 0.4 mL min^−1^, following a gradient starting with 95% B at 0.0 → 1.5 min, linear ramping down to 5% B at 2.5 → 6.5 min and then increased to 95% B at 8.0 min with a post-run time of 4.0 min (Kaczyński & Łozowicka, 2015). The mobile phases included for 2,4-D were 10 mM aqueous ammonium acetate (A) and methanol (B), and the gradient used was as described earlier (Meftaul et al., 2020b). The mobile phases for dimethoate were 10 mM aqueous ammonium formate (A) and methanol (B) (Utture et al., 2012; Meftaul et al., 2020c). The data obtained were processed using Agilent OpenLAB CDS ChemStation software. The standard curves were linear over the tested concentration range of glyphosate, 2,4-D and dimethoate with *R*^*2*^ values of 0.9958, 0.9980 and 0.9989, respectively. The values of LOD (limit of detection) and LOQ (limit of quantitation) obtained were 0.0039 and 0.0078 mg L^‒1^, respectively. The mean recoveries (*n* = 3) of spiked glyphosate, 2,4-D and dimethoate ranged from 0.0078 to 1.0 mg L^‒1^, and % recoveries were in the range of 83.84–101.01, 87.98–117.94 and 96.42–107.18, respectively.

LC-DAD system (Agilent Technologies, USA) with the detector wavelength set at 233 nm was used to quantify chlorothalonil from the aqueous samples (Báez et al., 2017). The mobile phase includes 0.01 M aqueous solution of phosphoric acid (A) and acetonitrile (B) with a flow rate of 0.50 mL min^‒1^. The injection volume was 30 μL with a gradient involving 90% B at 0.0 → 4.0 min, 98% B at 4.0 → 9.0 min, 20% B at 9.0 → 10.0 min, then 90% B at 10.0 → 12.0 min followed by a post-run for 1.0 min (Meftaul et al., 2021b). The data obtained were processed using Agilent OpenLAB CDS ChemStation software. A linear standard curve with a correlation coefficient (*R*^*2*^) of 0.9989 was obtained over the concentration range used. The LOD and LOQ values for chlorothalonil were 0.0078 and 0.0156 mg L^‒1^, respectively. The mean recoveries (*n* = 3) of chlorothalonil ranged from 0.0156 to 1.0 mg L^–1^, whereas the recoveries were in the range of 93.43–105.84%. Thus, the methods adopted here appeared to be reliable and accurate to quantify all four pesticides in different urban landscape soils.

### Data analysis

The rate of degradation of pesticides was calculated using the following equation:

1 $$ln{C}_{t}=-kt+ln{C}_{0}$$

where C_t_ and C_0_ are the amounts (mg kg^‒1^) of pesticide remaining in soil at a given time t and zero, respectively, and *k* is the degradation rate constant (day^‒1^). The DT_50_, which represents the time (days) needed for 50% disappearance of the initial amount of pesticide, was calculated from *k* using the equation (Hiller et al., 2012):

2 $${DT}_{50}=\frac{ln2}{k}$$

#### Environmental health risk assessment

The groundwater ubiquity score (GUS) and leachability index (LIX) were calculated using the Eqs. (3) and (4), respectively (Hall et al., 2015; Martins et al., 2018; Peruchi et al., 2015; Spadotto, 2002).

3 $$\mathrm{GUS}={\mathrm{logt}}^{1/2}\left(4-\mathrm{log}Koc\right)$$

4 $$LIX=\mathrm{exp}(-k \times Koc)$$

5 $$Koc=\frac{{K}_{d}}{\%OC}\times 100$$

where DT_50_ is the half-life (days) of pesticide in soil, *k* is the degradation rate constant (day^−1^) and *K*oc is the coefficient of organic carbon (L g^−1^) in soil. *K*_d_ is the solid–aqueous phase distribution coefficient (Hall et al., 2015).

#### Human non-cancer risk assessment

The potential non-cancer health risk for adults and children was determined following the widely adopted methods of USEPA (2020). The exposure pathway of pesticide from contaminated soil via ingestion was used while considering human health risk. The non-dietary chronic daily intake (CDI_i_) of pesticide (mg kg^−1^ day^−1^) via the incidental ingestion of the contaminated soil in adults and children was calculated based on the following equations (Bhandari et al., 2020).

6 $${\mathrm{CDI}}_{\mathrm{i}}=\frac{{C}_{s}\times EF\times ED\times {IR}_{i}}{AT \times BW} \times CF$$

where C_s_ (mg kg^−1^) is the concentration of pesticide residue in the soil after 50% degradation, EF is the exposure frequency (days yr^−1^), ED is the exposure duration (yrs), IR_i_ is the rate of contaminated soil ingestion (mg day^−1^), AT is the average lifetime (days), BW is the average body weight (kg) and CF is the conversion factor (kg mg^−1^).

7 $${\mathrm{CDI}}_{\mathrm{d}}=\frac{{C}_{s}\times DA\times DAF\times AF\times EF\times ED}{AT \times BW} \times CF$$

where CDI_d_ is the estimated non-dietary CDI (mg kg^‒1^ day^‒1^) of pesticide-contaminated soil particles via dermal contact, DA is the exposed dermal area (cm^2^ day^‒1^), DAF is the dermal adherence factor (mg cm^‒2^) for soil and AF (dimensionless) is the dermal absorption factor.

8 $${\mathrm{CDI}}_{\mathrm{ih}}=\frac{{C}_{\mathrm{s}}\times EF\times ED\times {IR}_{ih}}{PEF \times AT \times BW}$$

where CDI_ih_ is the estimated non-dietary CDI (mg kg^−1^ day^‒1^) of pesticide-contaminated soil particles via inhalation pathway, IR_ih_ is the rate of inhalation (m^3^ day^‒1^) and PEF is the particle emission factor (m^3^ kg^‒1^).

The non-cancer risk of pesticides is expressed as hazard quotient (HQ), whereas hazard index (HI) is the sum of HQ of individual pesticides, which was calculated following the equation (Afrin et al., 2021; Nisha et al., 2021; USEPA, 2020):

9 $$\mathrm{HQ}=\frac{CDI}{RfD}$$

10 $$\mathrm{HI}=\sum {HQ}_{Pesticide}$$

where RfD is the reference dose (mg kg^‒1^ day^‒1^) of a pesticide. The maximum acceptable reference doses (RfDs) in humans for glyphosate, 2,4‒D, chlorothalonil and dimethoate considered are 1 × 10^‒1^, 1 × 10^‒2^, 1.5 × 10^‒2^ and 2 × 10^‒4^ mg kg^‒1^ day^‒1^, respectively (MMDH, 2017; OEHHA, 2017a, 2017b; USEPA, 2016; USEPA, 1987a, 1987b).

### Statistical analysis

The experimental data obtained were processed using Microsoft Excel (Excel 2016). To establish the degree of correlation (*P* < 0.05) between multiple soil properties (predictors) and DT_50_ values of four pesticides in five urban soils, multivariate analysis was carried out using JMP pro 14/2021 software. Principle component analysis (PCA) was performed to determine the potential linear relationships (*P* < 0.05) between environmental parameters, soil *K*_d,_ GUS, LIX and DT_50_ values of the pesticides.

## Results and discussion

### Degradation of selected pesticides in urban landscape soils

**Fig. 1:**
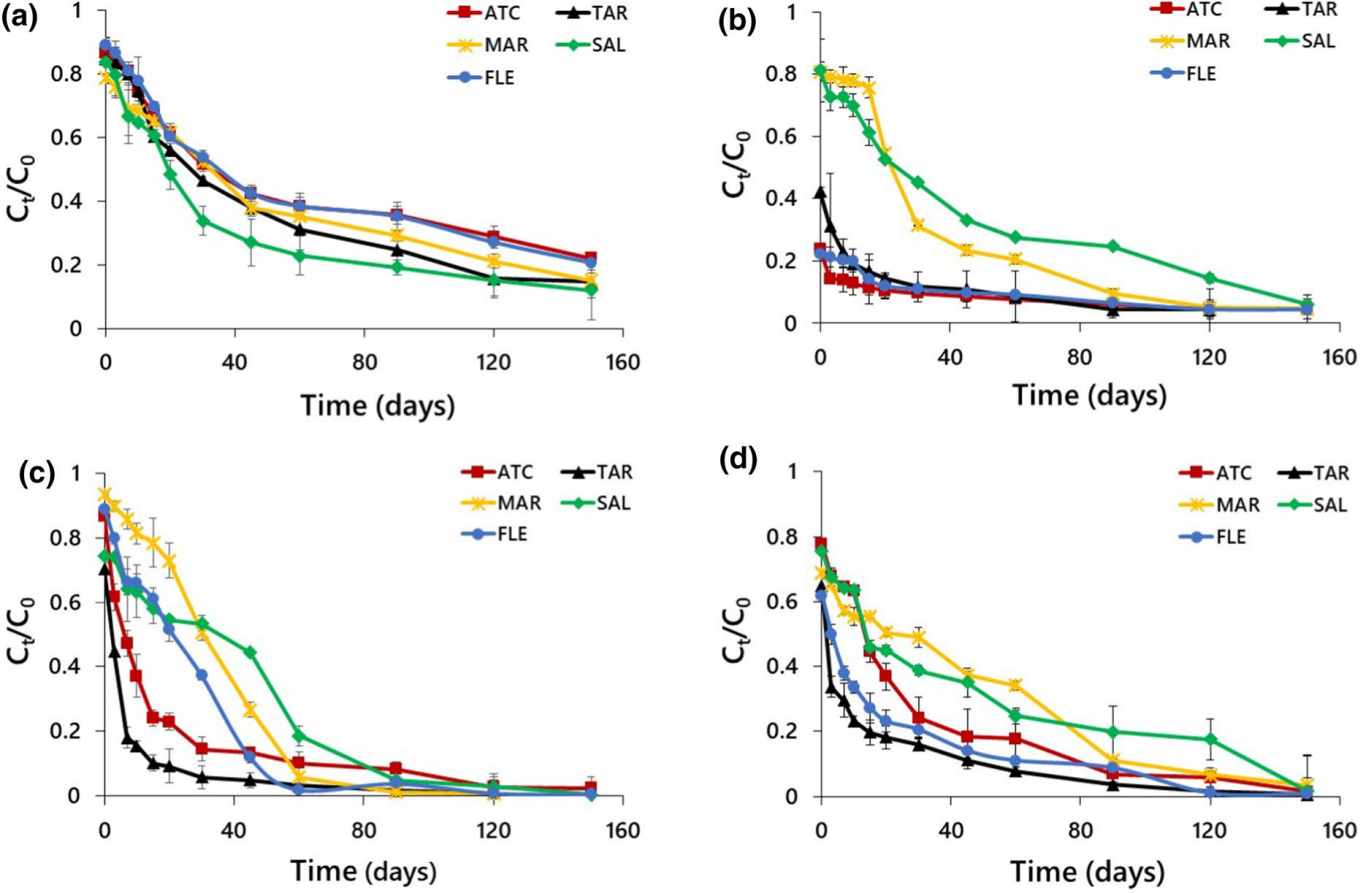
*C*_*t*_/*C*_0_ *vs* degradation time of (**a**) glyphosate, (**b**) 2,4-D, (**c**) chlorothalonil and (**d**) dimethoate in five urban landscape soils

The data on pesticide degradation rate constant, *k* (day^‒1^), and calculated DT_50_ values of four pesticides (two herbicides, one insecticide and one fungicide) in five urban landscape soils are presented in Fig. 1 and Table 1. Degradation rate constants of glyphosate, 2,4-D, chlorothalonil and dimethoate in the selected urban soils were in the range of 0.009 (ATC)–0.041 (MAR). The values of coefficient of determination (*R*^*2*^) for the pesticides in five soils were in the range of 0.908–0.982, 0.871–0.971, 0.901–0.960 and 0.843–0.977 for glyphosate, 2,4-D, chlorothalonil and dimethoate, respectively (Table S2). The calculated DT_50_ values, which can vary with the environmental conditions, soil depth and microbial activities (Oliveira et al., 2013), for glyphosate, 2,4-D, chlorothalonil and dimethoate in five urban landscape soils were in the range of 53–78, 32–75, 17–32 and 24–36, respectively (Table 1). Thus, the half-life of pesticides in soils tested followed the order: glyphosate > 2,4-D > dimethoate > chlorothalonil.

**Table 1:** Environmental and human health risk assessment of four pesticides in five urban landscape soils based on experimental half‒life (DT_50_) and pesticide residues after 50% degradation

Pesticide	Soil ID	*k *(day^‒1^)	*K*_d_	DT_50_ (days)	GUS	LIX	Adults			Children		
							HQ_i_	HQ_d_	HQ_ih_	HQ_i_	HQ_d_	HQ_ih_
Glyphosate	ATC	0.009	2.25	78	1.98	0.01	1.40 × 10^‒5^	7.30 × 10^‒6^	1.81 × 10^‒9^	7.27 × 10^‒6^	1.98 × 10^‒6^	9.36 × 10^‒10^
	TAR	0.012	2.23	56	3.42	0.25	1.54 × 10^‒5^	8.00 × 10^‒6^	1.98 × 10^‒9^	7.97 × 10^‒6^	2.17 × 10^‒6^	1.02 × 10^‒10^
	MAR	0.011	2.24	64	1.76	˂0.01	1.25 × 10^‒5^	6.52 × 10^‒6^	1.61 × 10^‒9^	6.49 × 10^‒6^	1.77 × 10^‒6^	8.36 × 10^‒10^
	SAL	0.013	3.10	53	1.57	˂0.01	1.60 × 10^‒5^	8.32 × 10^‒6^	2.06 × 10^‒9^	8.28 × 10^‒6^	2.26 × 10^‒6^	1.06 × 10^‒10^
	FLE	0.010	2.33	73	3.25	0.18	1.40 × 10^‒5^	7.30 × 10^‒6^	1.81 × 10^‒9^	7.27 × 10^‒6^	1.98 × 10^‒6^	9.36 × 10^‒10^
2,4‒D	ATC	0.009	3.03	75	4.50	0.69	1.60 × 10^‒4^	8.35 × 10^‒5^	2.07 × 10^‒8^	8.31 × 10^‒5^	2.27 × 10^‒5^	1.07 × 10^‒8^
	TAR	0.017	3.89	40	2.75	0.04	1.40 × 10^‒4^	7.27 × 10^‒5^	1.80 × 10^‒8^	7.24 × 10^‒5^	1.97 × 10^‒5^	9.32 × 10^‒9^
	MAR	0.022	3.93	32	1.10	˂0.01	1.56 × 10^‒4^	8.11 × 10^‒5^	2.01 × 10^‒8^	8.08 × 10^‒5^	2.20 × 10^‒5^	1.03 × 10^‒8^
	SAL	0.016	2.95	44	1.53	˂0.01	1.49 × 10^‒4^	7.77 × 10^‒5^	1.92 × 10^‒8^	7.74 × 10^‒5^	2.11 × 10^‒5^	9.96 × 10^‒9^
	FLE	0.011	2.43	61	3.09	0.12	1.61 × 10^‒4^	8.40 × 10^‒5^	2.08 × 10^‒8^	8.36 × 10^‒5^	2.28 × 10^‒5^	1.07 × 10^‒8^
Chlorothalonil	ATC	0.022	5.95	32	3.18	0.19	1.04 × 10^‒4^	5.40 × 10^‒5^	1.34 × 10^‒8^	5.38 × 10^‒5^	1.47 × 10^‒5^	6.93 × 10^‒9^
	TAR	0.029	7.55	24	1.96	˂0.01	1.02 × 10^‒4^	5.33 × 10^‒5^	1.32 × 10^‒8^	5.31 × 10^‒5^	1.44 × 10^‒5^	6.83 × 10^‒9^
	MAR	0.041	1.19	17	1.53	˂0.01	5.88 × 10^‒5^	3.05 × 10^‒5^	7.57 × 10^‒9^	3.04 × 10^‒5^	8.30 × 10^‒6^	3.91 × 10^‒9^
	SAL	0.032	1.37	22	1.68	˂0.01	9.79 × 10^‒5^	5.08 × 10^‒5^	1.26 × 10^‒8^	5.06 × 10^‒5^	1.38 × 10^‒5^	6.51 × 10^‒9^
	FLE	0.036	1.72	19	2.41	0.01	8.25 × 10^‒5^	4.28 × 10^‒5^	1.06 × 10^‒8^	4.26 × 10^‒5^	1.16 × 10^‒5^	5.48 × 10^‒9^
Dimethoate	ATC	0.024	3.21	29	3.47	0.36	7.38 × 10^‒3^	3.83 × 10^‒3^	9.50 × 10^‒7^	3.81 × 10^‒3^	1.04 × 10^‒3^	4.91 × 10^‒7^
	TAR	0.028	4.10	25	2.37	0.01	6.46 × 10^‒3^	3.35 × 10^‒3^	8.32 × 10^‒7^	3.34 × 10^‒3^	9.12 × 10^‒4^	4.29 × 10^‒7^
	MAR	0.020	1.41	35	2.99	0.10	8.11 × 10^‒3^	4.21 × 10^‒3^	1.04 × 10^‒6^	4.19 × 10^‒3^	1.14 × 10^‒3^	5.39 × 10^‒7^
	SAL	0.020	1.95	36	1.72	˂0.01	7.58 × 10^‒3^	3.93 × 10^‒3^	9.75 × 10^‒7^	3.91 × 10^‒3^	1.06 × 10^‒3^	5.04 × 10^‒7^
	FLE	0.029	3.02	24	2.28	0.01	8.27 × 10^‒3^	4.29 × 10^‒3^	1.06 × 10^‒6^	4.27 × 10^‒3^	1.16 × 10^‒3^	5.50 × 10^‒7^

*k*, Degradation rate constant; *K*_d_, Solid-aqueous phase distribution coefficient; DT_50,_ Calculated half-life; GUS; Groundwater ubiquity score; LIX, Leachability index; HQ, Hazard quotient via ingestion (HQ_*i*_), dermal (HQ_*d*_) and inhalation (HQ_*ih*_) pathways

The present results of DT_50_ values are in conformity with the half-lives reported for glyphosate (Meftaul et al., 2020d), 2,4-D (Jote, 2019), chlorothalonil (Van Scoy & Tjeerdema, 2014) and dimethoate (Martikainen, 1996). The slower degradation rate and longer DT_50_ values observed for glyphosate and 2,4-D might be due to their higher sorption capacity to soil minerals or dissolved OC limiting the access of pesticides to microbial degradation. The degradation of pesticides could be largely affected by the extent of soil organic matter content (Marin-Benito et al., 2012). However, some organic amendments may decrease the degradation rate of pesticides by augmenting the sorption capacity (Fernandes et al., 2006; Rodríguez-Cruz & Lacorte, 2005), whereas some of them favour degradation through stimulating microbial activity (Kadian et al., 2008; Marin-Benito et al., 2012). In contrast, the rapid degradation rate of chlorothalonil and dimethoate associated with shorter DT_50_ values could be due to their lower sorption capacity to soil matrix (Dhareesank et al., 2005). Generally, pesticides are known to bind to soil particles immediately after the entry or dissolve in soil solution and then desorb making them readily available for microbial degradation (Kočárek et al., 2018). The reason for relatively weak/irreversible bonding of pesticides and their availability in soil solution for biodegradation could be due to the occurrence of undecomposed or partially decomposed OC that was observed floating in the centrifuge tubes. In some cases, pesticides sorbed on to dissolved OC could increase the bioavailability and consequent biodegradation (Marin-Benito et al., 2012).

### Impact of urban soil properties on pesticide degradation

Degradation or transformation of a pesticide is strongly influenced by its bioavailability and physicochemical properties of soil (Singh et al., 2006). Some pesticides are strongly bound to soil particles, become unavailable for biodegradation and persist for extended periods in soil (Purnomo et al., 2011; Wang et al., 2013). The degradation rate constant, *k* (day^−1^) of glyphosate, 2,4-D, chlorothalonil and dimethoate in five urban soils was inversely proportional to the content of soil organic carbon (OC). For instance, slower degradation followed by higher DT_50_ values of these pesticides were observed in soils ATC, TAR and FLE that contained higher amounts of OC (7.66, 2.02 and 1.29%, respectively) as compared to other two soils (MAR and SAL) (Table S1). Also, the presence of higher amounts of clay in soils ATC, TAR and FLE was found to be the reason for decreased rate of pesticide degradation and higher DT_50_ values. Thus, soil OC together and clay content seem to be the predominant factors that play a pivotal role in pesticide degradation rate in urban soils since bound residues are not readily available for further transport and degradation (Koskinen et al., 2001). These observations clearly corroborate with those reported for agricultural soils by Purnomo et al. (2011) and Wang et al. (2013). Though the selected urban soils exhibited varying levels (0.21–7.66%) of OC (Table S1), the presence of undecomposed or partially decomposed OC that was floating in the centrifuge tubes could be the fact for almost similar degradation rates of pesticides in the tested soils. Moreover, it has been well established that pesticides are strongly bound to well-decomposed organic carbon making them unavailable for biodegradation (Ren et al., 2018).

The reason for strong binding of pesticides to OC observed in the selected soils might be the occurrence of varying functional groups such as O–H, C = O, C–H and C = C (Fig. S2) that have electrostatic/covalent/H bonds. In particular, C = O and C–H groups are more reactive and are implicated in solubility, cation exchange, polarity, chemical reactivity and wettability (Meftaul et al., 2021a, 2021b). Furthermore, the negatively charged clay minerals consist of tetrahedral silicate and octahedral aluminate groups that might react with pesticides via electrostatic interaction or cation exchange. Pesticide molecules become immobilized with soil clay minerals by forming surface complexes with metal ions (Barja & dos Santos, 2005). The extent of pesticide degradation was significantly less, followed by an increase in DT_50_ values of pesticides in soils containing higher amounts of silt, and Fe and Al oxides. The soils TAR, ATC and FLE that contained higher amounts of silt (55, 41.20 and 23.80%, respectively) showed a slower rate of pesticide degradation and prolonged DT_50_ values when compared with soils MAR and SAL having 16.20 and 1.20% silt (Table S1). The rate of pesticide degradation was low in soils that contained higher amounts of Fe and Al oxides in the clay fraction (Okada et al., 2020) since negatively charged pesticides molecules have strong affinity towards transition metals to form complexes in soil solutions (Barja & dos Santos, 2005). Thus, the degradation rate of pesticides was slower in soils that contained higher amounts of OC, clay, silt and oxides of Fe and Al, while OC and clay were the significant contributors.

The urban landscape soils used in the present study had similar types of minerals, while quartz was the predominant mineral constituent among albite, zeolite, sodalite, dolomite, orthoclase, hyalophane, etc., and their role on degradation of pesticides was almost the same. In contrast, urban soils exhibited higher pH (slightly alkaline) with more sand that increased the degradation rate of pesticides. In fact, soils with higher pH decrease the partition of pesticides molecules probably due to electrostatic repulsion caused by the presence of more net negative surface charges of soil minerals (Okada et al., 2020). In most cases, the degradation rate was slower in soil ATC having pH 5.8, and faster degradation was observed in soil MAR with pH 8.0. The pesticide degradation rate was higher in soils SAL and MAR with 97.60 and 76.30% sand, whereas it was slower in soil ATC having 51.30% sand (Table S1).

**Fig. 2:**
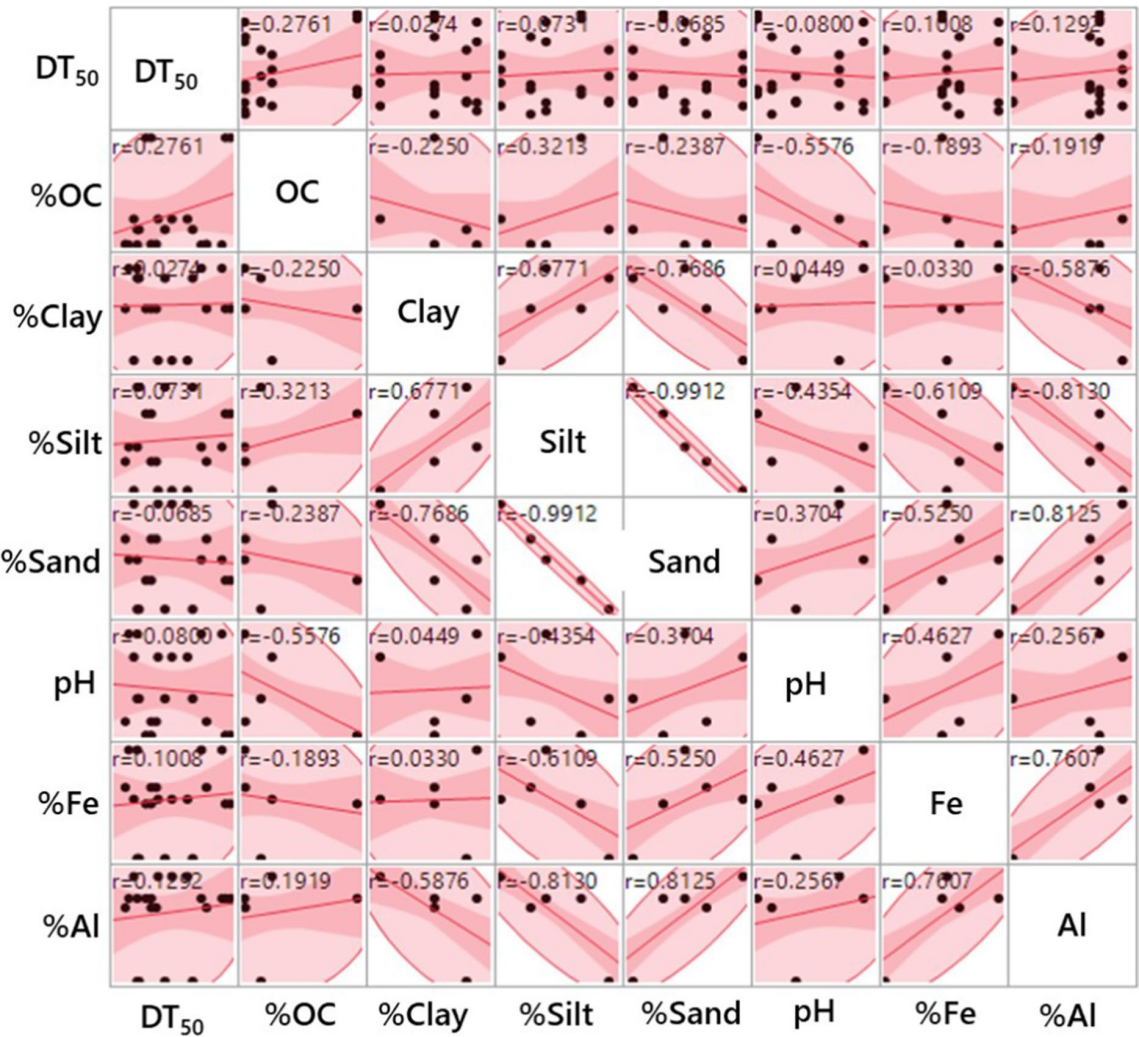
Relationship between soil properties (predictors), and half-life (DT_50_) values (outcome variable) of four pesticides in five urban landscape soils

The data on multivariate analysis, performed to further establish the interactions between soil characteristics (predictors) and DT_50_ values of pesticides, are presented in Fig. 2. Among all the predictors tested, OC (*R*^*2*^ = 0.276), %clay (*R*^*2*^ = 0.027), %silt (*R*^*2*^ = 0.073) and oxides of Fe (*R*^*2*^ = 0.101) and Al (*R*^*2*^ = 0.129) exhibited a significant positive correlation (*P* < 0.05) with DT_50_ values (*R*^*2*^ = 1.0) of all the selected pesticides. In contrast, soil pH (*R*^*2*^ = ‒0.08, *P* < 0.05) and sand content (*R*^*2*^ = ‒0.069) showed a negative correlation (*P* < 0.05) towards DT_50_ values of the pesticides. These observations indicate that the degradation of pesticides in soils was affected by several characteristics, including soil OC, clay and silt content, oxides of Fe and Al, and soil pH. However, the soil parameters showed a statistically significant negative correlation with the rate of pesticide degradation in most cases. Our finding clearly suggests that most of the soil properties are inversely correlated with the degradation rate and positively correlated with the observed DT_50_ values of pesticides.

### Environmental health risk assessment

Pesticide contamination of water bodies is a growing global concern because it affects non-target organisms, drinking water quality and food safety. Assessing the leaching potential is crucial while considering the environmental risk of pesticides, which is determined by *K*_oc_ based on *K*_d_ values. Using the values of DT_50_ and *K*_oc_, the environmental indices such as GUS and LIX were calculated, and the data are presented in Table 1. The *K*_d_ values determined for glyphosate, 2,4-D, chlorothalonil and dimethoate in the selected urban soils were in the range of 1.19–5.95 (Table 1), while the *K*oc values ranged from 39.57 to 1870.37 (Table S2).

The range of calculated GUS values for glyphosate, 2,4-D, chlorothalonil and dimethoate was 1.57–3.42, 1.10–4.50, 1.53–3.18 and 1.72–3.47, respectively (Table 1). Generally, soils could be considered as leachers, transitional and non-leachers if the GUS values are > 2.80, 1.80–2.80 and < 1.80, respectively (Martins et al., 2018; Meftaul et al., 2020c). Thus, soils ATC is considered as transitional, TAR and FLE soils are the potential leachers, and soils SAL and MAR are the non-leachers for glyphosate. For 2,4-D, soils ATC and FLE are the potential leachers, while soil TAR is the transitional, and soils MAR and SAL are the non-leachers. In case of chlorothalonil, soil ATC is the potential leachers, while soils TAR and FLE are the transitional, and MAR and SAL are the non-leachers. Soils ATC and MAR are the potential leachers, soils TAR and FLE are the transitional, and SAL is the non-leacher for dimethoate. Thus, our findings indicate the high leaching potential of all the four pesticides in urban soils, posing potential environmental hazards through contamination of water sources.

The calculated LIX values for glyphosate, 2,4-D, chlorothalonil and dimethoate were in the range of ˂0.01‒0.25, ˂0.01‒0.69, ˂0.01‒0.19 and ˂0.01‒0.36, respectively (Table 1). Generally, LIX values vary between 0.0 and 1.0, which indicate the least and profuse leaching potential, respectively (Martins et al., 2018; Meftaul et al., 2020a). Accordingly, leaching potential of the tested urban soils followed the order: TAR > FLE > ATC > SAL > MAR for glyphosate; ATC > FLE > TAR > SAL > MAR for 2,4-D; ATC > FLE > TAR > SAL > MAR for chlorothalonil; and ATC > MAR > FLE > TAR > SAL for dimethoate. These findings indicate the moderate to least leaching potential of the selected pesticides, which might pose environmental hazards by contaminating both surface and groundwater reservoirs. Likewise, the calculated values of both GUS and LIX clearly suggest that the pesticides leach into water sources from soil surface in urban landscapes and pose a potential threat to the health of aquatic organisms and other non-target biota.

**Fig. 3:**
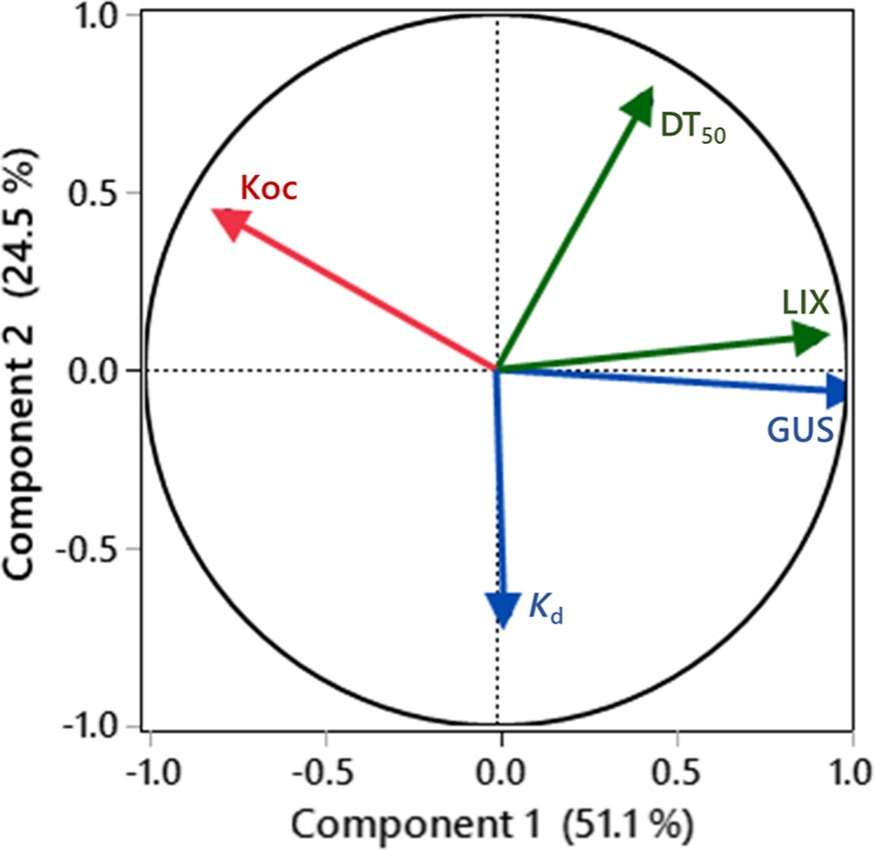
PCA score plots showing relationship among environmental parameters and half-life (DT_50_) values of four pesticides in five urban landscape soils

To further establish the interaction effects of multiple environmental parameters (predictors) and DT_50_ values of pesticides, principal component analysis (PCA) was performed and the results are presented in Fig. 3. PCA is an important multivariate analysis that converts the bulk of data input variables to some common factors that are correlated (Shahid et al., 2020). Among all the predictors, *K*oc (*R*^*2*^ = 0.083), GUS (*R*^*2*^ = 0.365) and LIX (*R*^*2*^ = 0.403) exhibited a significant positive correlation (*R*^*2*^ = 1.0, *P* < 0.05) with DT_50_ values of pesticides. The PCA converted the whole data into four major component factors. The corresponding variance values for the predictors and DT_50_ values were 24.51, 17.60, 6.33 and 0.48%, respectively. The calculated eigenvalues for the above predictors were 1.22, 0.88, 0.32 and 0.03, respectively (Fig. 3). The first two principal components were attributed to approximately 51.08% of the cumulative variance with an eigenvalue of 2.55. In contrast, *K*_d_ values negative correlated (*R*^*2*^ = ‒0.389, *P* < 0.05) with DT_50_ values of pesticides.

### Human health risk assessment

Health risk for adults and children, in terms of non-cancer ailments associated with the exposure to pesticide-contaminated soil, was evaluated using widely adopted equations and constant parameters (Table 2). The data on non-dietary chronic daily intake (CDI) of pesticide from contaminated soils through ingestion, dermal and inhalation pathways and well-established USEPA models were used to calculate the potential non-cancer risks in adults and children caused by pesticide exposure. The non-cancer risk of pesticide exposure through a pathway is denoted as the hazard quotient (HQ), which is the ratio of the estimated average non-dietary CDI value and the RfD of a contaminant (Brum et al., 2021). The data obtained for non-cancer risk associated with the exposure to pesticide-contaminated urban soils after 50% degradation are presented in Table 1 and Table S2. The calculated values of non-dietary CDI (mg kg^‒1^ day^‒1^) for adults upon exposure to pesticide-contaminated urban soils through ingestion, dermal and inhalation pathways for the four pesticides were significantly higher than those for children (Table S2). Similarly, the HQ values determined for glyphosate, 2,4-D, chlorothalonil or dimethoate exposure of adults via ingestion, dermal and inhalation pathways in five urban soils were significantly higher than those observed for children (Table 1). On the other hand, the HI values of pesticides in five urban landscape soils obtained for adults via ingestion, dermal and inhalation pathways were significantly higher than those for children (Table S3). If the value of HQ or HI is < 1, it indicates that the people exposed to pesticides are safe, whereas a value > 1 indicates a non-cancer health risk (Bhandari et al., 2020; Parven et al., 2021). The HQ and HI values for the selected four pesticides in five urban soils were several times lower than the recommended threshold values of HQ and HI. Our present data demonstrate that human exposure to pesticide residues after 50% degradation in urban soils through ingestion, dermal and inhalation pathways would cause extremely low or unnoticeable non-cancer risks for adults and children. Human non-cancer risk estimates of pesticide residues in urban soils were also lower than those reported in Central India by Kumar et al. (2018), in Novi Sad, Serbia, by Škrbić et al. (2017) and in Nepal by Bhandari et al. (2020). However, there could be a health hazard to pets and children if exposed immediately after pesticide application to the ornamental plants, lawns and parks in urban landscapes (Meftaul et al., 2021a). To our knowledge, this is the first comprehensive study that investigated the possible hazards of human and environmental health associated with four pesticides used extensively in urban landscapes.

**Table 2:** Constant parameters and their values for the estimation of non‒carcinogenic risk in adults and children (based on data from Bhandari et al., 2020)

S.no.	Exposure factor	Human adults	Children
1	Ingestion rate (IR_*i*_)	100 mg day^‒1^	100 mg day^‒1^
2	Inhalation rate (IR_*ih*_)	17.50 m^3^ day^‒1^	17.50 m^3^ day^‒1^
3	Body weight (BW)	62 kg	12 kg
4	Averaging lifetime (AT)	70 years (25,550 days)	70 years (25,550 days)
5	Exposure frequency (EF)	350 days yr^‒1^	350 days year^‒1^
6	Exposure duration (ED)	30 years	3 years
7	Exposed dermal area (DA)	5700 cm^2^ day^‒1^	1050 cm^2^ day^‒1^
8	Dermal adherence factor (DAF)	0.07 mg cm^‒2^	0.20 mg cm^‒2^
9	Dermal absorption factor (AF)	0.13 mg cm^‒2^	0.13 mg cm^‒2^
10	Particle emission factor (PEF)	1.36 × 10^9^ m^3^ kg^‒1^	1.36 × 10^9^ m^3^ kg^‒1^
11	Conversion factor (CF)	1 × 10^‒6^ kg mg^‒1^	1 × 10^‒6^ kg mg^‒1^

## Conclusion

The current novel study determined the values of degradation rate constant, *k*, and DT_50_ of four pesticides in five urban landscape soils to establish the associated human and environmental health risks. The *k* values of four pesticides significantly correlated with soil properties: a positive correlation with pH and sand content and a negative correlation with OC, clay, silt and oxides of Fe and Al. On the contrary, the calculated DT_50_ values of four pesticides in urban soils were positively correlated with OC, clay, silt and oxides of Fe and Al, while a negative correlation was evident with soil pH and sand content. The environmental risk assessment, in terms of GUS and LIX indices of glyphosate, 2,4-D, chlorothalonil and dimethoate indicated the portability of pesticides from the soil surface to water bodies that might affect non-target biota. Human non-cancer risk of pesticides, based on calculated values of HQ and HI indices for adults and children via ingestion, dermal and inhalation pathways, suggested that exposure to pesticide-contaminated soils, after 50% degradation, might cause zero or negligible non-carcinogenic risks. To minimize the exposure risks and safeguard the environmental and human health, improved formulations with microbially derived pesticides should be applied in urban landscapes. In addition, precision band spraying might limit the pesticide usage and its transport besides avoiding the build-up of resistant target organisms.

## Supplementary Information

Below is the link to the electronic supplementary material.

Supplementary file1 (DOCX 590 KB)

## Data Availability

The datasets used in this study are available from the corresponding author on reasonable request.
