# Correction to: Degradation of four pesticides in five urban landscape soils: human and environmental health risk assessment

**DOI:** 10.1007/s10653-022-01314-9

**Published:** 2022-06-25

**Authors:** Islam Md Meftaul, Kadiyala Venkateswarlu, Prasath Annamalai, Aney Parven, Mallavarapu Megharaj

**Affiliations:** 1 Global Centre for Environmental Remediation (GCER), College of Engineering, Science and Environment, The University of Newcastle, ATC Building, University Drive, Callaghan, NSW 2308 Australia; 2 Department of Agricultural Chemistry, Sher-e-Bangla Agricultural University, Dhaka, 1207 Bangladesh; 3 Formerly Department of Microbiology, Sri Krishnadevaraya University, Anantapuramu, 515003 India; 4 Cooperative Research Centre for Contamination Assessment and Remediation of the Environment (CRC CARE), The University of Newcastle, Callaghan, NSW 2308 Australia

## Correction to: Environ Geochem Health 10.1007/s10653-022-01278-w

In the original publication of the article, the Table 1 and Electronic Supplementary Material was incorrect.

The article has been updated with the correct Table 1 and Electronic Supplementary Material.

The original article has been corrected.

