# Supplementary material for: Degradation of four pesticides in five urban landscape soils: human and environmental health risk assessment
Source: Environ Geochem Health. 2022 May 11;45(5):1599–614. doi: 10.1007/s10653-022-01278-w (PMC10140087; doi:10.1007/s10653-022-01278-w)
Supplement: Supplementary file 1 — Supplementary file1 (DOCX 590 KB) [file 10653_2022_1278_MOESM1_ESM.docx]

**Supplementary Information**

**Degradation of four pesticides in five urban landscape soils: Human and environmental health risk assessment**

**Islam Md Meftaul • Kadiyala Venkateswarlu • Prasath Annamalai • Aney Parven** **• Mallavarapu Megharaj**^*^

**I. M. Meftaul • P. Annamalai • A. Parven • M. Megharaj**

Global Centre for Environmental Remediation (GCER), College of Engineering, Science and Environment, The University of Newcastle, Callaghan, NSW 2308, Australia

**I. M. Meftaul • A. Parven**

Department of Agricultural Chemistry, Sher‒e‒Bangla Agricultural University, Dhaka‒1207, Bangladesh

**K. Venkateswarlu**

Formerly Department of Microbiology, Sri Krishnadevaraya University, Anantapuramu 515003, India

**M. Megharaj**

Cooperative Research Centre for Contamination Assessment and Remediation of the Environment (CRC CARE), The University of Newcastle, Callaghan, NSW 2308

****Address for correspondence***:

**Mallavarapu Megharaj**

Global Centre for Environmental Remediation (GCER)

College of Engineering, Science and Environment

The University of Newcastle, ATC Building

University Drive, Callaghan, NSW 2308, Australia

Mobile: +61 411126857; orcid.org/0000‒0002‒6230‒518X

E‒mail: megh.mallavarapu@newcastle.edu.au

**
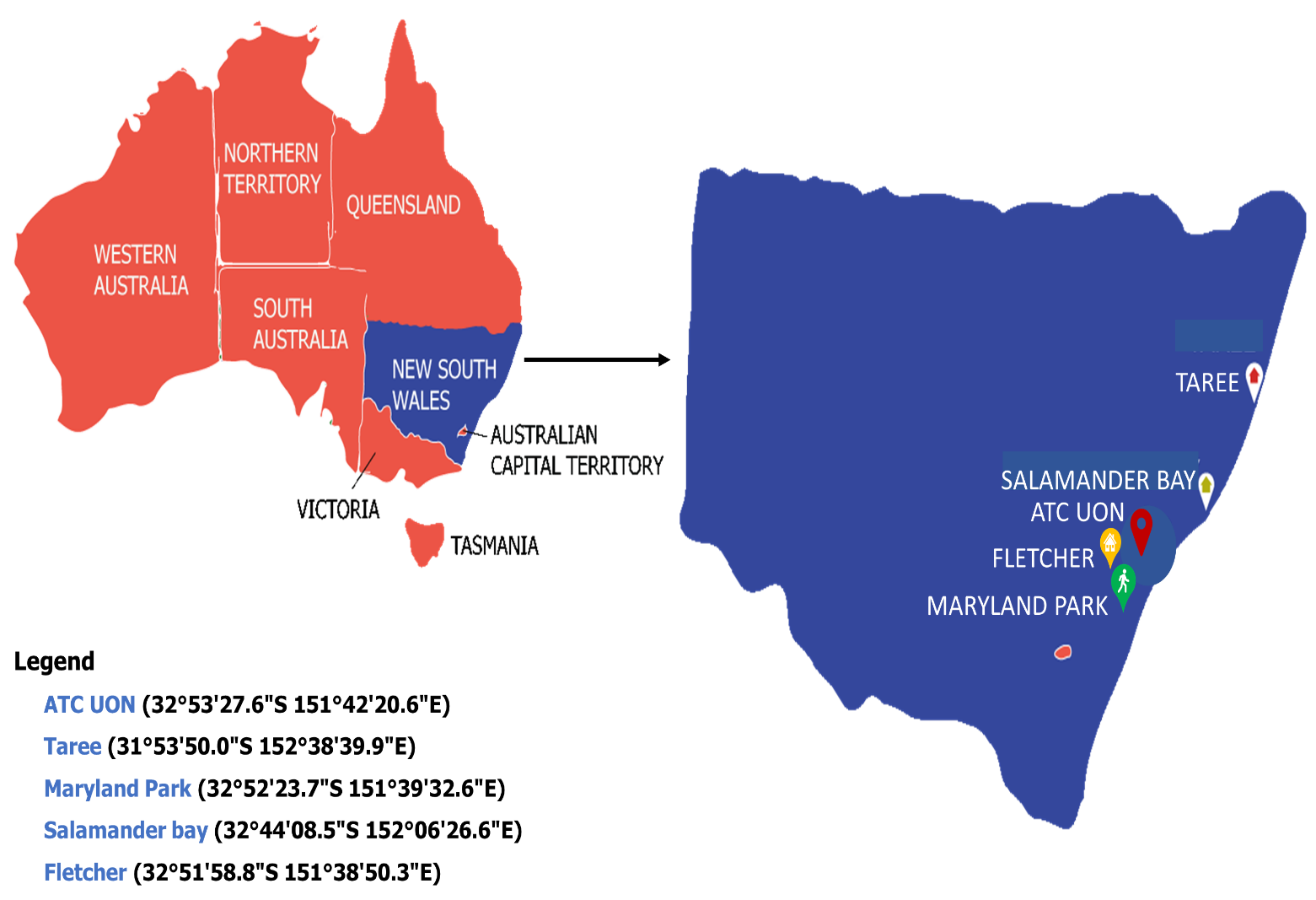
**

**Fig. S1** Map showing different sampling locations in New South Wales, Australia.

**
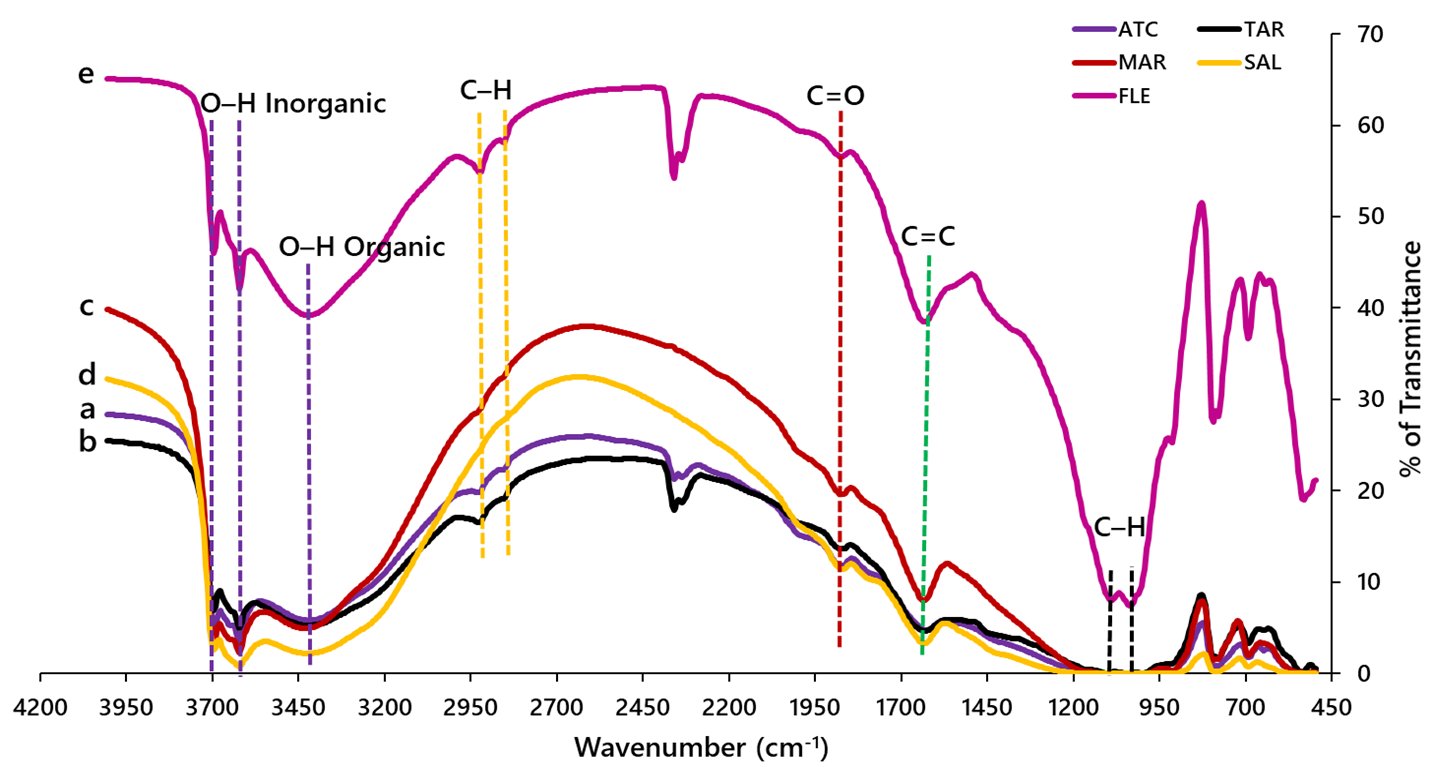
**

**Fig. S2** Fourier‒transform infrared (FTIR) spectra indicating the functional groups present in the selected urban soils.

**Table S1** Physicochemical properties of five soils collected from the urban landscapes.

| Soil ID | Location | OC^a^ (%) | Fe^b^  (%) | Al^b^  (%) | Sand (%) | Silt (%) | Clay (%) | pH^c^  (in Milli‒Q water) |  | Textural  class | Major mineral compound |
| --- | --- | --- | --- | --- | --- | --- | --- | --- | --- | --- | --- |
| ATC | Lawn | 7.66 ±0.01 | 1.10±0.09 | 0.73±0.11 | 51.30 | 41.20 | 7.50 | 5.8±0.03 |  | Loam | Quartz, Orthoclase, Albite, Hyalophane |
| FLE | Home garden | 1.29 ±0.02 | 0.03±0.67 | 0.01±0.99 | 63.80 | 23.80 | 12.40 | 6.6±0.03 |  | Sandy loam | Quartz, Oligoclase, Albite, Sodalite |
| SAL | Home garden | 0.25 ±0.16 | 1.42±0.02 | 0.65±0.15 | 97.60 | 1.20 | 1.20 | 6.1±0.02 |  | Sand | Quartz, Dolomite, Zeolite LC‒3, Palladium |
| TAR | Home garden | 2.02 ±0.01 | 1.19±0.03 | 0.92±0.11 | 33.80 | 55 | 11.20 | 7.5±0.03 |  | Silt loam | Quartz, Sinnerite, Ice Ic, Sylvine, Bernalite, Albite |
| MAR | Park | 0.21 ±0.10 | 2.15±0.03 | 0.73±0.18 | 76.30 | 16.20 | 7.50 | 8.0±0.01 |  | Loamy sand | Quartz, Albite, Zeolite, Sodalite |

^a^Analyzed in LECO analyser (LECO Corporation, USA). ^b^Analyzed in ICP‒OES (Perkin Elmer Pvt Ltd, Singapore) after microwave digestion (MARS 6™, USA). ^c^Determined in a pH meter (Horiba Scientific, UK). ATC = University of Newcastle; FLE = Fletcher; SAL = Salamander Bay; TAR = Taree; MAR = Maryland.

**Table S2** Concentration of pesticide residue in the soil after 50% degradation **(**C_s_), coefficient of organic carbon (*K*_oc_), coefficient of determination (*R^2^*) and estimated average non-dietary chronic daily intake (CDI), in terms of mg kg^−1^ day^−1^, via ingestion (CDI_i_), dermal (CDI_d_) and inhalation (CDI_ih_) pathways of four pesticides for adults and children in five urban landscape soils.

| Pesticide | Soil ID | C_s_  (mg kg^‒1^) | *K*_OC_  (L g^−1^) | *R^2^* | Adults | | | Children | | |
| --- | --- | --- | --- | --- | --- | --- | --- | --- | --- | --- |
|  |  |  |  |  | CDI_i_ | CDI_d_ | CDI_ih_ | CDI_i_ | CDI_d_ | CDI_ih_ |
| Glyphosate | ATC | 2.13 | 901.78 | 0.938 | 1.40×10^‒6^ | 7.30×10^‒7^ | 1.81×10^‒10^ | 7.27×10^‒7^ | 1.98×10^‒7^ | 9.36×10^‒11^ |
|  | TAR | 2.33 | 110.57 | 0.960 | 1.54×10^‒6^ | 8.00×10^‒7^ | 1.98×10^‒10^ | 7.97×10^‒7^ | 2.17×10^‒7^ | 1.02×10^‒10^ |
|  | MAR | 1.90 | 1066.05 | 0.982 | 1.25×10^‒6^ | 6.52×10^‒7^ | 1.61×10^‒10^ | 6.49×10^‒7^ | 1.77×10^‒7^ | 8.36×10^‒11^ |
|  | SAL | 2.42 | 1240.34 | 0.908 | 1.60×10^‒6^ | 8.32×10^‒7^ | 2.06×10^‒10^ | 8.28×10^‒7^ | 2.26×10^‒7^ | 1.06×10^‒10^ |
|  | FLE | 2.13 | 180.27 | 0.948 | 1.40×10^‒6^ | 7.30×10^‒7^ | 1.81×10^‒10^ | 7.27×10^‒7^ | 1.98×10^‒7^ | 9.36×10^‒11^ |
| 2,4‒D | ATC | 2.43 | 39.57 | 0.871 | 1.60×10^‒6^ | 8.35×10^‒7^ | 2.07×10^‒10^ | 8.31×10^‒7^ | 2.27×10^‒7^ | 1.07×10^‒10^ |
|  | TAR | 2.12 | 192.57 | 0.881 | 1.40×10^‒6^ | 7.27×10^‒7^ | 1.80×10^‒10^ | 7.24×10^‒7^ | 1.97×10^‒7^ | 9.32×10^‒11^ |
|  | MAR | 2.36 | 1870.37 | 0.960 | 1.56×10^‒6^ | 8.11×10^‒7^ | 2.01×10^‒10^ | 8.08×10^‒7^ | 2.20×10^‒7^ | 1.03×10^‒10^ |
|  | SAL | 2.26 | 1181.56 | 0.971 | 1.49×10^‒6^ | 7.77×10^‒7^ | 1.92×10^‒10^ | 7.74×10^‒7^ | 2.11×10^‒7^ | 9.96×10^‒11^ |
|  | FLE | 2.44 | 188.26 | 0.921 | 1.61×10^‒6^ | 8.40×10^‒7^ | 2.08×10^‒10^ | 8.36×10^‒7^ | 2.28×10^‒7^ | 1.07×10^‒10^ |
| Chlorothalonil | ATC | 2.36 | 77.67 | 0.907 | 1.56×10^‒6^ | 8.11×10^‒7^ | 2.01×10^‒10^ | 8.07×10^‒7^ | 2.20×10^‒7^ | 1.03×10^‒10^ |
|  | TAR | 2.33 | 373.72 | 0.901 | 1.54×10^‒6^ | 7.99×10^‒7^ | 1.98×10^‒10^ | 7.96×10^‒7^ | 2.17×10^‒7^ | 1.02×10^‒10^ |
|  | MAR | 1.33 | 567.06 | 0.960 | 8.82×10^‒7^ | 4.58×10^‒7^ | 1.13×10^‒10^ | 4.56×10^‒7^ | 1.24×10^‒7^ | 5.87×10^‒11^ |
|  | SAL | 2.22 | 546.16 | 0.960 | 1.46×10^‒6^ | 7.62×10^‒7^ | 1.89×10^‒10^ | 7.59×10^‒7^ | 2.07×10^‒7^ | 9.76×10^‒11^ |
|  | FLE | 1.87 | 133.40 | 0.921 | 1.23×10^‒6^ | 6.42×10^‒7^ | 1.59×10^‒10^ | 6.39×10^‒7^ | 1.74×10^‒7^ | 8.23×10^‒11^ |
| Dimethoate | ATC | 2.23 | 41.85 | 0.971 | 1.47×10^‒6^ | 7.66×10^‒7^ | 1.90×10^‒10^ | 7.63×10^‒7^ | 2.08×10^‒7^ | 9.82×10^‒11^ |
|  | TAR | 1.95 | 202.78 | 0.977 | 1.29×10^‒6^ | 6.70×10^‒7^ | 1.66×10^‒10^ | 6.68×10^‒7^ | 1.82×10^‒7^ | 8.59×10^‒11^ |
|  | MAR | 2.45 | 116.26 | 0.973 | 1.62×10^‒6^ | 8.42×10^‒7^ | 2.08×10^‒10^ | 8.38×10^‒7^ | 2.29×10^‒7^ | 1.07×10^‒10^ |
|  | SAL | 2.29 | 779.63 | 0.843 | 1.51×10^‒6^ | 7.86×10^‒7^ | 1.95×10^‒10^ | 7.83×10^‒7^ | 2.13×10^‒7^ | 1.00×10^‒10^ |
|  | FLE | 2.49 | 234.05 | 0.950 | 1.65×10^‒6^ | 8.58×10^‒7^ | 2.13×10^‒10^ | 8.55×10^‒7^ | 2.33×10^‒7^ | 1.10×10^‒10^ |

**Table S3** Hazard index (HI) values for human adults and children via ingestion (HI_i_), dermal (HI_d_), and inhalation (HI_ih_) pathways based on the sum of HQ of individual pesticides in five urban landscape soils.

| Soil ID | Adults | | | Children | | |
| --- | --- | --- | --- | --- | --- | --- |
|  | HI_i_ | HI_d_ | HI_ih_ | HI_i_ | HI_d_ | HI_ih_ |
| ATC | 7.66×10^–3^ | 3.97×10^–3^ | 9.86×10^–7^ | 3.96×10^–3^ | 1.08×10^–3^ | 5.09×10^–7^ |
| TAR | 6.72×10^–3^ | 3.48×10^–3^ | 8.65×10^–7^ | 3.47×10^–3^ | 9.48×10^–4^ | 4.47×10^–7^ |
| MAR | 8.34×10^–3^ | 4.32×10^–3^ | 1.07×10^–6^ | 4.31×10^–3^ | 1.17×10^–3^ | 5.54×10^–7^ |
| SAL | 7.84×10^–3^ | 4.06×10^–3^ | 1.00×10^–6^ | 4.05×10^–3^ | 1.10×10^–3^ | 5.21×10^–7^ |
| FLE | 8.53×10^–3^ | 4.42×10^–3^ | 1.09×10^–6^ | 4.41×10^–3^ | 1.20×10^–3^ | 5.67×10^–7^ |
